# Enabling Mg metal anodes rechargeable in conventional electrolytes by fast ionic transport interphase

**DOI:** 10.1093/nsr/nwz157

**Published:** 2019-10-21

**Authors:** Ruijing Lv, Xuze Guan, Jiahua Zhang, Yongyao Xia, Jiayan Luo

**Affiliations:** 1 Key Laboratory for Green Chemical Technology of Ministry of Education, State Key Laboratory of Chemical Engineering, School of Chemical Engineering and Technology, Tianjin University, Tianjin 300072, China; 2 Department of Chemistry, Fudan University, Shanghai 200433, China

**Keywords:** fast ion transport interphase, artificial layer, Mg metal anodes, conventional electrolytes

## Abstract

Rechargeable magnesium batteries have received extensive attention as the Mg anodes possess twice the volumetric capacity of their lithium counterparts and are dendrite-free. However, Mg anodes suffer from surface passivation film in most glyme-based conventional electrolytes, leading to irreversible plating/stripping behavior of Mg. Here we report a facile and safe method to obtain a modified Mg metal anode with a Sn-based artificial layer via ion-exchange and alloying reactions. In the artificial coating layer, Mg_2_Sn alloy composites offer a channel for fast ion transport and insulating MgCl_2_/SnCl_2_ bestows the necessary potential gradient to prevent deposition on the surface. Significant improved ion conductivity of the solid electrolyte interfaces and decreased overpotential of Mg symmetric cells in Mg(TFSI)_2_/DME electrolyte are obtained. The coated Mg anodes can sustain a stable plating/stripping process over 4000 cycles at a high current density of 6 mA cm^−2^. This finding provides an avenue to facilitate fast ion diffusion kinetics of Mg metal anodes in conventional electrolytes.

## INTRODUCTION

Over the past few decades, lithium-ion batteries (LIBs) have been widely used as energy storage systems owing to their safety and long lifespan. However, the limited energy density of LIBs and depletion of lithium resources cannot meet the increasing demand [1]. Hence, there is an urgent need to explore new battery systems with high energy density and reduced cost. The recent emergence of rechargeable magnesium batteries (RMBs) has received growing attention due to several inherent strengths. Metallic Mg possesses a low redox potential (−2.3 V vs. standard hydrogen electrode), high abundance in the earth’s crust and quite a high theoretical volumetric capacity of 3833 mAh cm^−3^, twice that of Li [1–3]. The high volumetric capacity and low cost promote the practical applications of RMBs for large-scale energy storage systems and electric vehicles [4,5]. Furthermore, Mg is less sensitive to air and moisture than Li, and its dendrite-free nature upon cycling eliminates the safety concerns of cell internal short circuits [6,7]. However, one fatal obstacle for RMBs is the formation of ionic insulating surface passivation film on Mg anodes in most conventional electrolytes [8–13], leading to irreversible plating/stripping behavior of Mg.

Tremendous efforts on various electrolyte systems have been devoted to inhibiting the formation of ionic insulating interfaces. Preliminary literature has demonstrated that Grignard solutions enable sustainable plating/stripping of metallic magnesium [14,15]. In 2000, Aurbach’s group first proposed that more stable and highly efficient cycling can be realized by modifying the Lewis acid–base reactions between the magnesium and aluminum reagents [16]. But the nucleophilic characteristic of these carbanions may limit the usage of high-capacity electrophilic cathodes (e.g. sulfur) [17–19]. Subsequently, research toward exploring non-nucleophilic electrolytes with high anodic stability has been stimulated. Numerous achievements have been attained regarding the development of non-nucleophilic electrolytes for RMBs, including all phenyl complexes (APC) [20,21], hexamethyldisilazide (HMDS)-based electrolytes [22,23], magnesium aluminum chloride complex (MACC) electrolytes [24–27], borohydride [28–30] electrolytes, boron-cluster [31,32] and glyme-based electrolytes [8,10,33]. Other than electrolyte studies, work concerning Mg anode modification in conventional electrolytes has been rare. Very recently, Son *et al*. reported an artificial Mg^2+^-conducting polymeric interphase engineered on the Mg powder surface, which facilitated a markedly reversible Mg plating/stripping process in Mg(TFSI)_2_-based electrolytes [9]. But caution needs to be taken during processing of reactive Mg powder.

**Figure 1.**
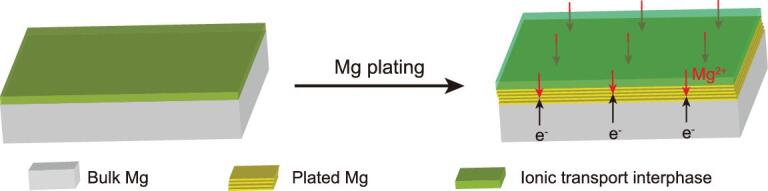
Schematic showing the Mg plating behavior in conventional Mg(TFSI)_2_/DME electrolytes with ionic transport interphase on Mg metal anodes.

Previous works have demonstrated that Sn and Mg_2_Sn electrodes were compatible with conventional electrolytes for rechargeable Mg-ion batteries, capable of higher capacities and lower insertion/extraction voltages [34]. However, the anodes undergo huge volume fluctuation caused by de-/alloy reactions during charge/discharge cycling. Herein we propose a facile, safe and effective approach to prepare modified Mg metal anodes with artificial layers, which exhibit fast Mg^2+^ diffusion. The approach was realized by direct reduction of SnCl_2_ by Mg foils at room temperature. The resultant Sn reacts with the underlying Mg to give a film composed of Mg^2+^ conducting tin-based compounds (e.g. Mg_2_Sn, Sn, Fig. S1), and electron insulating MgCl_2_/SnCl_2_ are formed as by-products of the ion-exchange reaction. Previous works have demonstrated that Sn and Mg_2_Sn electrodes were compatible with conventional electrolytes for rechargeable Mg-ion batteries, with high capacity and low voltages [34]. The Sn-based artificial layer remains compositionally stable on cycling, providing a fast pathway for Mg^2+^ transport to the underlying Mg, and the nonconductive characteristic of MgCl_2_/SnCl_2_ prevents Mg deposition on the top surface (Fig. 1). The modified Mg metal anodes deliver an extended lifespan over 4000 cycles (1400 h) and low overvoltage of ∼0.2 V of repeated plating/stripping in symmetric cells at high rates of 6 mA cm^−2^ in conventional Mg(TFSI)_2_/DME electrolyte. Moreover, TiS_2_ cathodes can deliver 220 mAh g^−1^ in full cells paired with SnCl_2_-treated Mg anodes.

## RESULTS AND DISCUSSION

**Figure 2.**
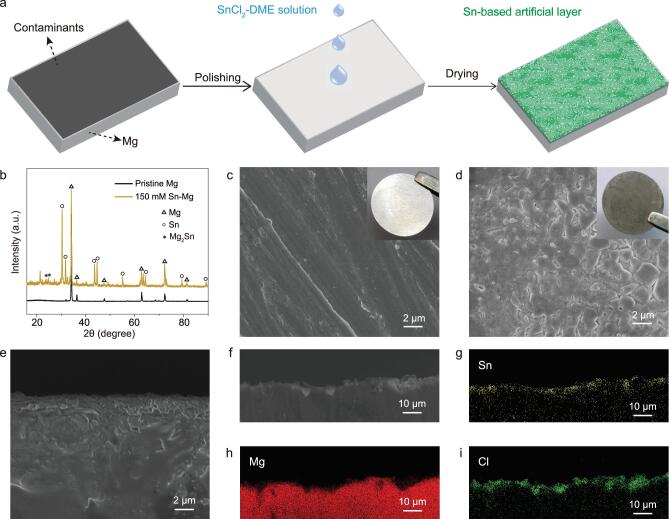
Synthesis and characterization of the artificial layer coating on Mg anodes. (a) Schematic illustration of the formation procedure of the modified Mg foils. (b) XRD patterns of pristine and modified Mg metal electrodes. SEM images of (c) pristine Mg metal electrodes and (d) modified Mg metal electrodes. Inset graphs are photos of pristine and modified Mg foils, respectively. (e) Cross-section SEM image of modified Mg metal electrodes. EDS mapping of (g) Sn, (h) Mg, and (i) Cl of (f) the cross section of modified Mg electrodes.

The facile Mg metal pretreatment methodology is shown in Fig. 2a. Mg foils were first polished with sandpaper until the surface was shiny to clean surface contaminants. A significant reduced content of oxide layer can be obtained for polished Mg electrode, confirmed by the results of scanning electron microscopy (SEM) and energy dispersive X-ray spectroscopy (EDS) (Fig. S2). Then, 100 μL of SnCl_2_-DME solution with different concentrations was dropped on the Mg surface and the reaction was allowed to proceed for several minutes until a dark gray coating was obtained via the simple ion exchange reaction: Mg + SnCl_2_ → Sn + MgCl_2_ [1]; *x*Mg + *y*Sn → Mg*_x_*Sn*_y_* [2]. The modified Mg foil was dried in an argon-filled glovebox before use. The structure and chemistry of the modified Mg metal anodes were characterized by X-ray diffraction (XRD) analysis (Fig. 2b), indicating that crystalline Mg_2_Sn and metallic Sn are formed on the surface of Mg electrodes. The extra peaks in the XRD patterns were assigned to SnO*_x_*, MgO*_x_* (Fig. S3) [35,36], which is inevitable when exposed to air during the XRD characterization. SEM was employed to confirm the morphologies of the pristine and modified Mg foils. Compared with pristine Mg foil (Fig. 2c), a uniform coating layer with a textured surface is presented for the modified one (Fig. 2d). The thickness of the alloy composite layer is about 2 μm as measured by cross-sectional SEM (Fig. 2e). Furthermore, EDS mapping of the modified electrode reveals a uniform distribution of Sn and Cl in the coating layer (Fig. 2f–i). It should be noted that the structure and electrochemical property of alloy-modified electrodes are concentration dependent. Thus, we prepared a set of SnCl_2_-DME solutions containing 50 mM, 100 mM and 150 mM Sn salt to observe the difference of their chemistries and morphologies and analyze the Mg^2+^ diffusion process of these modified interfaces. In the XRD pattern (Figs 2b, S4a), peaks at 30.3, 31.7, 43.5 and 44.6 degrees start to appear when the concentration is 50 mM, suggesting that Sn is formed. Increasing the salt concentration to 150 mM, the Mg_2_Sn phase is presented, which is evidenced by the peaks labeled with asterisks. Additionally, SEM images show that Sn-based artificial layers become denser and flatter with increasing Sn salt content (Figs 2d, S4b, c).

**Figure 3.**
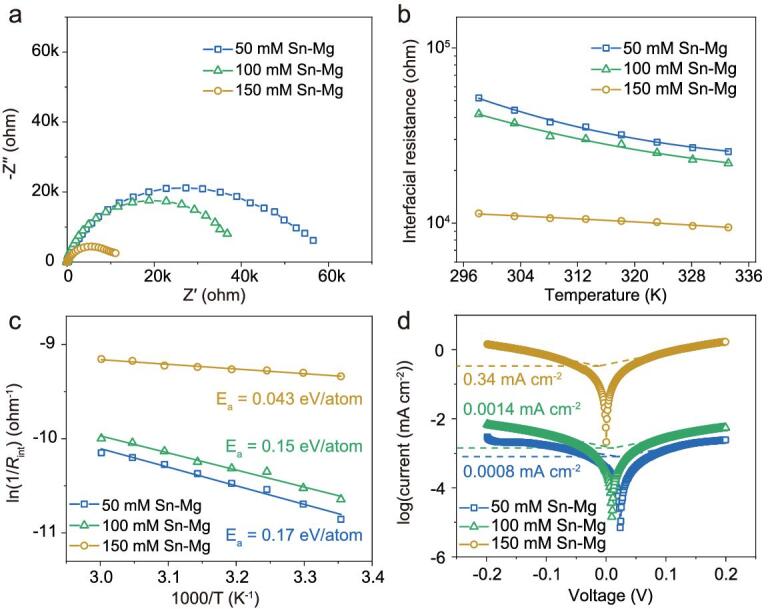
Electrochemical analysis of symmetric cells with SnCl_2_-treated Mg anodes. (a) Impedance spectra of symmetric cells with modified Mg anodes at room temperature treated with 50, 100 and 150 mM of SnCl_2_, respectively. (b) Temperature-dependent interfacial resistance of symmetric cells with modified Mg anodes. (c) Napierian logarithm of reciprocal interfacial impedance as a function of reciprocal temperature; the lines are Arrhenius model fits. (d) Tafel plots obtained from cyclic voltammetry measurements.

Impedance spectroscopy could reflect the ion diffusion process at the solid electrolyte interface. Figure 3a compares Nyquist plots of symmetric Mg/Mg cells with modified Mg metal anodes in Mg(TFSI)_2_/DME electrolyte. To assess the interfacial transport of Mg^2+^ in the Sn-based artificial layers quantitatively, each spectrum was fitted to an equivalent circuit model (Fig. S5 and Table S1). The model consists of a bulk resistance (*R*_b_) and an interfacial resistance (*R*_int_), representing ion transport in the electrolyte and ion transport through the interface, respectively. It is well known that a passivation layer would form on the surface of Mg metal anodes in Mg(TFSI)_2_/DME electrolyte; therefore a large *R*_int_ is displayed for pristine Mg foil (approximately 450 000 Ω) (Fig. S6), whereas the *R*_int_ values are decreased significantly for modified Mg electrodes and the values of cells with artificial layers are gradually lowered with increasing concentration of Sn-bearing solutions. In particular, a nearly 45-fold decrease of the interfacial resistance is observed when the concentration reaches 150 mM, similar to the fast ion diffusion layer on lithium metal anodes [37–41]. However, the solution appears oversaturated when the concentration exceeds 150 mM (Fig. S7); hence this salt content is selected as the maximum value for further electrochemical analysis.

The temperature dependence of the interfacial resistance can be used to extract information about how the artificial layers alter the energy barrier for ion transport (Fig. 3b and c). As expected, the interfacial resistance decreases with increasing temperature (Fig. S8). The data are well described by the Arrhenius formula, *R*_int_^−1^ = *A* exp(*−E*_a_/*RT*), implying that the interfacial transport is thermally activated. Here *A* is the prefactor, *E*_a_ is the apparent activation energy for ion transport and *R* is the universal gas constant. It is observed that the apparent activation energy of the Sn-based interface decreases with increasing concentration of the Sn-bearing solution and the value drops to 0.043 eV atom^−1^ when the concentration reaches 150 mM. It can be seen that *E*_a_ for the artificial coating with a treated concentration of 150 mM is apparently lower than the others, which means that the transport of Mg^2+^ at any temperature is much faster in this interface. As previously reported, this result is consistent with the joint density-functional theory study (JDFT) analysis [42,43], which could be interpreted as showing that a low interfacial activation energy contributes to a low diffusion energy barrier for Mg^2+^ transport.

Cyclic voltammograms (CV) were measured with modified/pristine Mg working electrode (WE) using a three-electrode system. In contrast to no noticeable peak being observed for the pristine Mg WE, the modified Mg electrode displayed a pair of reduction/oxidation peaks related to Mg plating/stripping processes (Fig. S9). Moreover, the 0.5 M MgTFSI_2_/DME electrolyte with a modified Mg electrode system exhibited a better stability (∼2.5 V) on stainless steel (Fig. S10). To further investigate the ion transport kinetics of the plating/stripping process, the exchange current density was measured. The values here were calculated from Tafel plots, which were in turn obtained from the CV for symmetric Mg/Mg cells (Fig. S11). The results elucidate that the exchange current density for Mg plating/stripping of the modified electrode pretreated with 150 mM Sn salt (0.34 mA cm^−2^) is substantially higher than the rest, as shown in Fig. 3d. The fast charge transfer kinetics obtained from the exchange current density reinforces our conclusions from the impedance results that Sn-based interfaces facilitate fast interfacial kinetics. The Tafel slope is measured experimentally. Tafel analysis also gives Tafel slope values of 0.369 V, 0.292 V and 0.275 V for 50 mM Sn–Mg, 100 mM Sn–Mg and 150 mM Sn–Mg anodes, respectively. According to the Tafel slope equation *A* = 2.303*kT*/*e*α, where *k* is Boltzmann’s constant (1.381 × 10^−23^ J K^−1^), *T* is the absolute temperature (298.15 K), *e* is the elementary charge (1.602 × 10^−19^ C), and α is the charge transfer coefficient, the electron transfer number for 150 mM Sn–Mg anode was calculated to be 2.15, indicating a two-electron stripping/plating process, which is higher than that of 50 mM Sn–Mg anode (1.60) and 100 mM Sn–Mg anode (2.03). According to the above proofs and corresponding symmetric electrochemical performances (Fig. S12), 150 mM SnCl_2_-DME solution is selected for subsequent physical characterizations and electrochemical tests.

**Figure 4.**
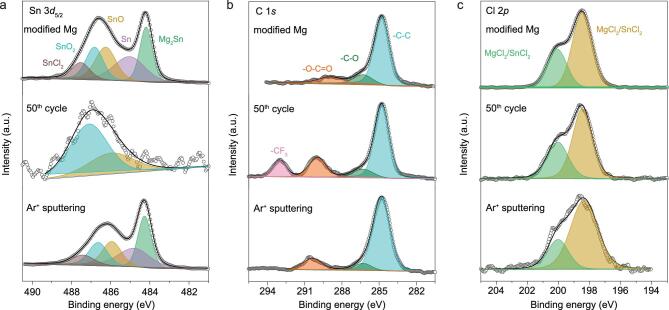
XPS analysis of the modified Mg metal anodes before and after cycling. (a) Sn 3*d*_5/2_, (b) C 1*s* spectra and (c) Cl 2*p* spectra of the artificial coating on Mg metal electrode, before cycling (top), and upon cycling for 50 cycles, before sputtering (center) and after 16 min Ar^+^ sputtering (bottom), respectively.

X-ray photoelectron spectroscopy (XPS) is conducted to further acquire the composition and stability upon cycling of the artificial layer (Fig. S13). As exhibited in Fig. 4a, the Sn 3*d*_5/2_ spectrum of Sn-treated Mg anode can be deconvoluted into several component peaks, consisting of Mg_2_Sn, Sn, SnO*_x_* and SnCl_2_. The peaks at 484.2 and 485.0 eV are assigned to Mg_2_Sn and Sn [44], respectively, which is consistent with the XRD pattern (Fig. 2b). The XPS spectra of C 1*s* can be fitted into three peaks centered at 284.5, 286.3 and 289.7 eV, which correspond to –C–C, –C–O and –O=C–O [45], respectively (Fig. 4b). The Cl 2*p* spectrum shows two split peaks of 2*p*_1/2_ and 2*p*_3/2_ at 200.4 and 198.7 eV, respectively, corresponding to MgCl_2_/SnCl_2_ [46,47] in the coating layer (Fig. 4c). The stability of Sn-based artificial layers is demonstrated by the XPS depth profiling analysis after 50 cycles, as shown in Fig. 4. The absence of Mg_2_Sn and Sn signals and the presence of the –CF_3_ functional group signal (293.2 eV) [48] imply that the very uppermost layer is covered by a buffer interphase caused by decomposition of electrolytes. The oxidation of Sn (SnO_2_, SnO) was the main component in the Sn 3*d*_5/2_ spectrum. Note that the total amount of Sn element on the anode surface after 50 cycles is merely 0.06 at% by XPS, which is quite small compared with that of the uncycled modified Mg anode (1.54 at%) (Fig. S13). Thus, the amount of SnO*_x_* on the surface of the artificial layer upon cycling for 50 cycles can be negligible. According to the XPS data, the ratio of the F and Mg elements of modified Mg anode after 50 cycles is 3.66, smaller than that of pristine Mg anode (7.67) after only 1 cycle (Fig. S14). Considering that the Mg content in the artificial layer is lower than pure Mg anode, the actual F product on the modified Mg anode should be much lower than that on the pristine Mg anode. This indicates that the solid electrolyte interphase (SEI) layer on the modified Mg anode is much thinner than that on the pristine Mg anode, which could allow Mg ions to pass through. After 16 min Ar^+^ sputtering, the predominance of Mg_2_Sn and Sn in the Sn 3*d*_5/2_ spectrum emerges; moreover, no signal of the –CF_3_ component is apparent in the C 1*s* spectrum, indicating that the thickness of the interphase is far less than the coating layer and electrolyte decomposition is restricted in this layer. It is noteworthy that MgCl_2_/SnCl_2_ is also evident in the Sn-based artificial layer before and after Ar^+^ sputtering, indicating that it penetrates well under the top surface during the preparation of modified Mg electrodes, consistent with the bulk probe provided by EDS (Fig. 2g–i). The surface stability of the artificial layer for long-term cycling is investigated as well. The XPS spectra confirm that the artificial layers of modified electrodes stay compositionally invariant after 150 cycles, resembling that after 50 cycles (Fig. S15).

**Figure 5.**
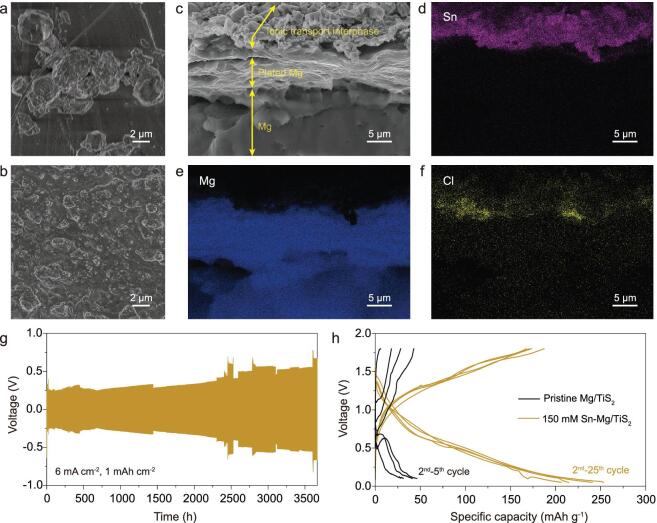
Electrochemical performance of the modified Mg metal anodes. Surface view of (a) the pristine Mg anode (0.05 mA cm^−2^) and (b) modified Mg anode (2 mA cm^−2^) plated with 2 mAh cm^−2^ of Mg. (c) Cross-sectional image for modified Mg anode plated with 2 mAh cm^−2^ and the corresponding EDS mapping of (d) Sn, (e) Mg, and (f) Cl, respectively. Voltage profiles in symmetric cells with modified Mg anodes at a current density of (g) 6 mA cm^−2^. (h) Galvanostatic voltage profiles of the pristine and modified Mg anodes paired with TiS_2_ cathode at a current density of 10 mA g^−1^.

We employed a combination of SEM and EDS mapping to investigate Mg electrodeposition with Sn-based artificial layers. Figure 5a shows a top-surface SEM view of the non-dendritic and particle-like morphology of electrodeposited magnesium on the pristine Mg electrode (0.05 mA cm^−2^, 2 mAh cm^−2^). When increased to a high rate of 2 mA cm^−2^, an irreversible plating/stripping behavior is exhibited for the pristine Mg anode (Fig. S16). The composition of the passivation film mainly consists of Mg (OH)_2_, MgO and organic components caused by the decomposition of electrolytes (Fig. S17). In contrast, the SnCl_2_-treated Mg electrode shows a relatively uniform and smooth Mg deposition (Fig. 5b). The cross-sectional view of the modified electrodes after electrodeposition with 2 mAh cm^−2^ of Mg shows that the laminar Mg is plated under the coating layer. The plated Mg layer appears bright in secondary electron detection mode (Fig. 5c), but dark in backscattered mode (Fig. S18b), allowing it to be distinguished from the Sn-based layer. The thickness of the deposited Mg is about 5 μm, in accordance with that expected for the plated quantity. Corresponding EDS mappings of Sn, Mg, Cl further testify to the location of the deposited Mg and coating layer (Fig. 5d–f).

Sn/Mg_2_Sn is indeed electrochemically active with both high electron and Mg^2+^ conductivity. But in our artificial layer, MgCl_2_/SnCl_2_ is not fully dissolved into the electrolyte, as shown in XPS. Sn/Mg_2_Sn is mixed with insulating MgCl_2_/SnCl_2_. As indicated by direct current–voltage measurements, the electronic resistivity of the coating layer is about 16 700 Ω cm and that of pristine Mg electrode about 45 Ω cm (Fig. S19). As a result, Sn/Mg_2_Sn acts as a Mg^2+^ conductor and MgCl_2_/SnCl_2_ act as an insulating component to establish the necessary potential gradient across the layer to drive Mg^2+^ diffusion through the film. In addition, a high diffusion coefficient *D*_Mg_ in the metallic tin bulk phase in the vicinity of 2.9 × 10^−11^ cm^2^ s^−1^ was reported [49], which far exceeds that of Mg in MgO (10^−20^ cm^2^ s^−1^) [50].

Stripping/plating measurements were carried out in symmetric Mg/Mg cells to validate the role of the Sn-based artificial layers of modified Mg anodes. A fair capacity of 0.005 mAh cm^−2^ at a current density of 0.01 mA cm^−2^ was continuously plated/stripped in each discharge/charge cycle. As shown in Fig. S10, the uncoated Mg electrode experiences extremely high and rapidly increasing overpotential (>2 V) upon cycles, caused by Mg surface passivation. The passivation layer quickly shuts off the passage of Mg^2+^, resulting in irreversible plating/stripping behaviors. In contrast, the SnCl_2_-treated Mg electrode exhibits better reversibility over 650 h without pronounced overpotential (∼0.2 V) during long-term cycles, which is mainly attributed to favorable ionic diffusion in the artificial layers. Similar galvanostatic discharge/charge experiments in Mg/Mg symmetric cells were performed under extremely high current densities of 6 mA cm^−2^. Figure 5g shows that the cells with modified electrodes exhibit narrow voltage gaps of ∼0.2 V and remain remarkably stable over 4000 cycles (1400 h). Note that all the cells with pristine Mg anodes could not exhibit sustainable plating/stripping, apparently caused by the Mg surface passivation layer as a result of electrolyte decomposition. In addition, a control experiment was conducted to exclude the effect of Cl^−^ dissolved in the electrolyte. As shown in Fig. S20, the cell with pristine Mg electrodes in Mg(TFSI)_2_–MgCl_2_/DME electrolyte still exhibits a large overpotential. After pretreating Mg metal with saturated SnF_2_/DME solution and 150 mM SnBr_2_/DME solution, the polarization voltage of the saturated SnF_2_–Mg electrode and 150 mM SnBr_2_–Mg electrode voltage are not further reduced in the chloride-containing electrolyte. This suggests that the reversible and stable Mg plating/stripping behavior is principally attributed to the Sn-based artificial layers. To our knowledge, this is the best electrochemical performance of Mg symmetric cells at high current densities compared to other studies that have employed Mg(TFSI)_2_/DME electrolytes. The performance of the SnCl_2_-treated Mg foil as an anode was further investigated in a Mg/TiS_2_ full cell. Due to the remarkably ionically conductive coating layer, the modified Mg/TiS_2_ full cell delivers a steady discharge capacity around 200 mAh g^−1^ and a low overvoltage after 25 cycles (Fig. 5h). As a comparison, rapid capacity fading accompanied by a sharp rise in overpotential is observed in the pristine Mg/TiS_2_ full cell, attributed to increasingly severe passivation of the Mg surface.

In conclusion, we have employed a simple ion-exchange chemistry to form a Sn-based artificial film with halide components at room temperature, providing a fast ion conduit for magnesium electrodeposition. The Sn-based compounds possess a high diffusion coefficient, enhancing diffusion kinetics in the bulk phase. In addition, the insulating nature of halides inhibits the deposition of magnesium on the surface and generates a driving force to induce magnesium to plate under the coating layer. Importantly, the artificial layer remains compositionally invariant upon continuous cycling. The ion-conductive film enables an ultralong lifespan over 4000 cycles (1400 h) with quite a low voltage gap of ∼0.2 V at high current densities of 6 mA cm^−2^. Stable cycling is realized by such modified anodes paired with a TiS_2_ cathode. Our strategy is significant in RMBs since it provides a promising avenue for generating a Mg^2+^-conducting film in conventional Mg(TFSI)_2_/DME electrolyte via a facile and viable method. We believe that this general approach will stimulate more research on modifying Mg anodes with other available Mg^2+^-conducting metals or alloys.

## METHODS

### Preparation of the modified Mg electrode

Electrode preparation was carried out in an argon-filled glovebox with < 0.1 ppm oxygen and moisture. SnCl_2_-DME solutions were prepared with different concentrations of 50 mM, 100 mM and 150 mM. Mg metal foils were thoroughly polished by sandpaper and rinsed with anhydrous ethanol to remove the surface oxide layer and expose a fresh Mg surface. Then the polished Mg foil was punched into disks with a diameter of 12 mm. After polishing, 100 μL of SnCl_2_-DME solution was dropped on the polished Mg foil surface and the reaction was allowed to proceed for a few minutes. Treated magnesium metal appeared dark gray on the surface and was dried in the glovebox before use. The preparation of the saturated SnF_2_–Mg electrode and 150 mM SnBr_2_–Mg electrode used the same procedure as the Sn–Mg electrode.

### Materials characterization

SEM (Hitachi S4800, Japan) was employed to characterize the morphology of the pristine and modified Mg. XRD patterns were recorded on a Rigaku D/max-2500B2+/PCX system with Cu Kα radiation. XPS spectra were collected on an SSI SProbe XPS spectrometer with an Al (Kα) source. The Ar^+^ sputtering rate was 6–7 nm min^−1^. XPS samples were sealed in a container filled with Ar and then transferred quickly to the XPS sample chamber to avoid sample exposure to air. The spectra were analyzed using software referenced to the C 1*s* binding energy of 284.6 eV. Different cycles of the Mg plating/stripping process on 150 mM Sn–Mg electrodes were conducted for the XPS and SEM characterization. The cells were disassembled in an argon-filled glovebox. After that, the electrodes were rinsed with DME and dried. Then the samples were sealed in a container and transferred quickly for characterization.

### Electrochemistry characterization

Electrochemical studies were performed using 2032 coin cells with symmetric designs. Symmetric Mg cells (pristine Mg foil or SnCl_2_-treated Mg foil with a diameter of 12 mm) were assembled with glass microfiber filters (Whatman, Grade GF/A) as the separator, and 100 μL of 0.5 M Mg(TFSI)_2_ in DME as the electrolyte. To test the performance in full cells, TiS_2_ was used as the cathode. TiS_2_ powder (Sigma Aldrich, −200 mesh, 99.9%) was stored in a glove box filled with Ar and used as received. The TiS_2_ electrode was prepared by mixing TiS_2_, Super P and polyvinylidene fluoride (PVDF) in the weight ratio of 7:2:1 in *N*-methyl pyrrolidone (NMP). Then the slurry was coated on stainless steel foil with a thickness of 10 μm and vacuum dried at 80°C for 12 h. The cathodes were cut into disks with a diameter of 12 mm. The loading mass of the active material in each piece was about 1 mg. Temperature-dependent impedance spectra were measured from 10 Hz [6] to 10^−2^ Hz at a temperature range of 25 to 60°C using an AC impedance spectroscopy instrument (Solartron Metrology). Cyclic voltammetry was performed on the Solartron Metrology with two identical electrodes at a sweep rate of 1 mV s^−1^. The electronic resistivity of the pristine and modified Mg electrodes was measured by examining the voltage response to a direct current on the cells with the pristine or modified Mg foils sandwiched between two stainless steel electrodes. The electrochemical operation window was measured by linear sweep voltammetry (LSV) on the SS/0.5 M Mg(TFSI)_2_-DME/150 mM Sn–Mg cell at a scan rate of 1 mV s^−1^. Three-electrode systems were carried out in a homemade glass cell with a total electrolyte volume of ∼5 mL in a typical experiment. The pristine/modified Mg electrode was used as the working electrode, the pristine/modified Mg electrode was used as the counter electrode, and Ag/AgCl was used as the reference electrode. Due to the low solubility of AgCl in glyme, the Ag/AgCl reference was instead formed by immersing the Ag wire in an aqueous sodium hypochlorite for a few minutes to form a thin AgCl layer on the wire surface.

## Supplementary Material

nwz157_Supplemental_File
