# Supplementary material for: Enabling Mg metal anodes rechargeable in conventional electrolytes by fast ionic transport interphase
Source: Natl Sci Rev. 2019 Oct 21;7(2):333–41. doi: 10.1093/nsr/nwz157 (PMC8288991; doi:10.1093/nsr/nwz157)
Supplement: nwz157_Supplemental_File [file nwz157_supplemental_file.docx]

**Supporting Information for**

**Enabling Mg Metal Anodes Rechargeable in Conventional Electrolytes by Fast Ionic Transport Interphase**

Ruijing Lv^1#^, Xuze Guan^1#^, Jiahua Zhang^1^, Yongyao Xia^2†^ and Jiayan Luo^1†^

^1^Key Laboratory for Green Chemical Technology of Ministry of Education, State Key Laboratory of Chemical Engineering, School of Chemical Engineering and Technology, Tianjin University, Tianjin 300072, China

^2^Department of Chemistry, Fudan University, Shanghai 200433, China

#Equally contributed to this work.

†Corresponding authors: E-mails: yyxia@fudan.edu.cn; [jluo@tju.edu.cn](mailto:jluo@tju.edu.cn)


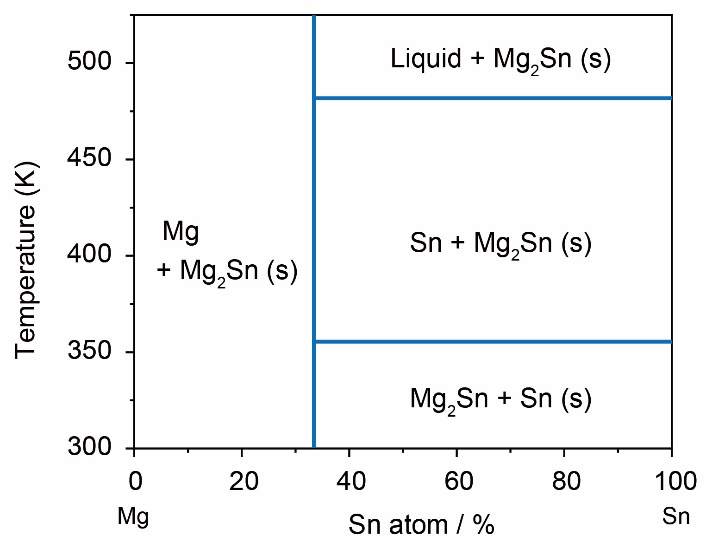


**Figure S1. Phase diagram of Mg-Sn alloy.**


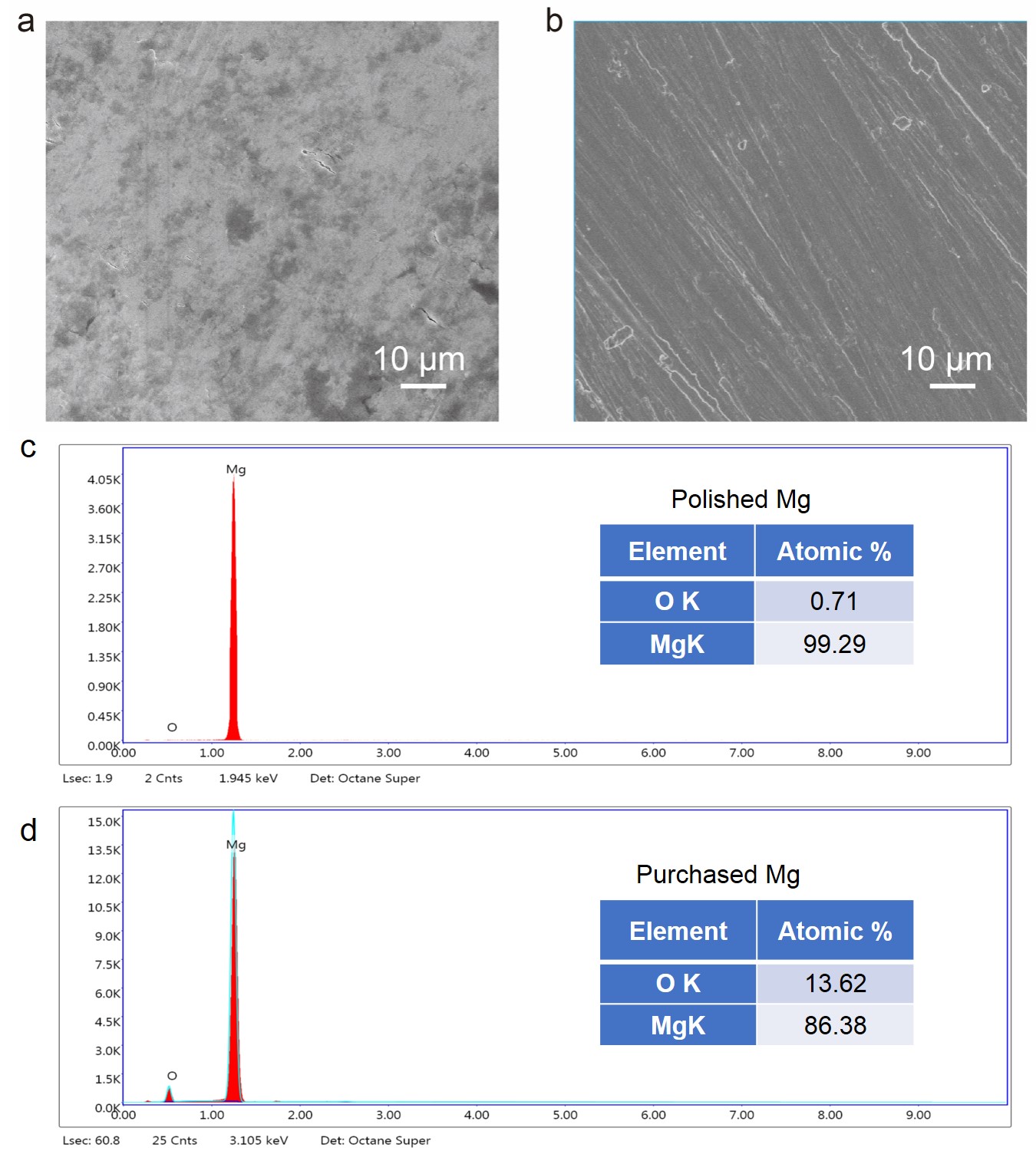


**Figure S2. SEM images and EDS analysis of purchased (a, d) and polished Mg foils (b, c).** The insulating property of oxide layer was exhibited a white area by SEM characterization for purchased Mg foils (Figure S2a). After polishing, the Mg metal exhibited intrinsic conductive property, as shown in Figure S2b. Therefore, a native oxide layer was largely removed after polishing. Furthermore, the results of EDS mapping also demonstrate that a reduced content of oxide layer could be obtained for polished Mg anode (Figure S2c, d).


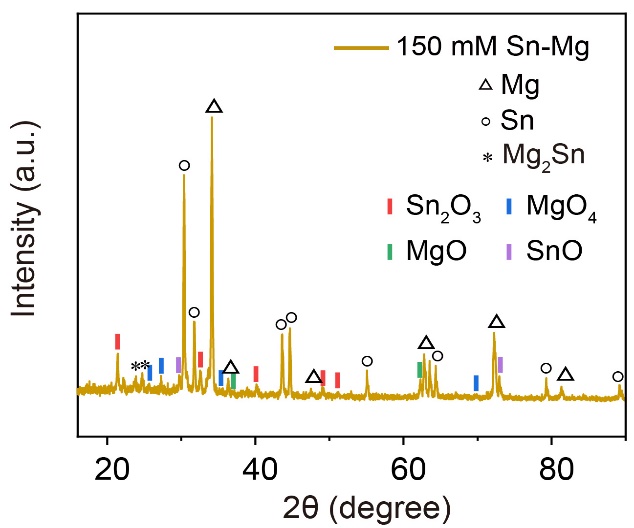


**Figure S3. XRD pattern of the modified Mg electrode pretreated with 150 mM SnCl_2_ solution.**


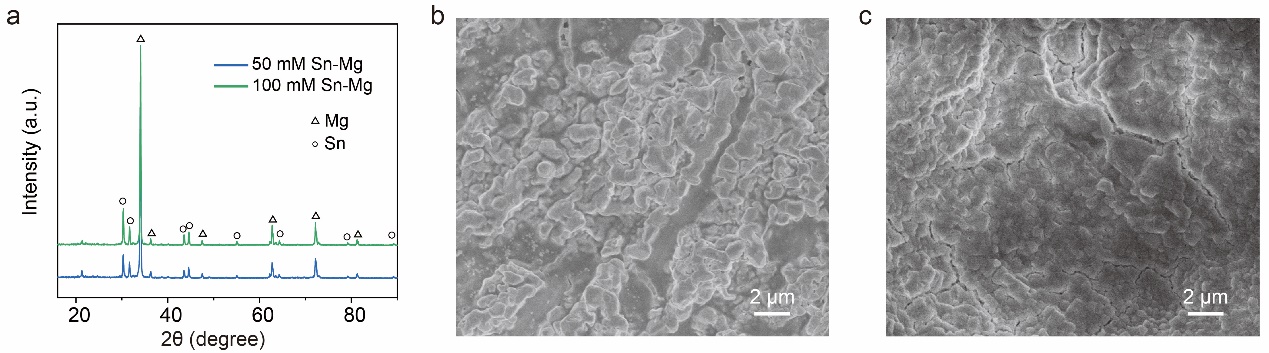


**Figure S4.** **Characterization of the artificial SEI layer coating on metallic Mg.** a) XRD profile of modified Mg prepared from the SnCl_2_ solution with concentrations of 50 mM and 100 mM, respectively. SEM images of (b) 50 mM Sn-Mg and (c) 100 mM Sn-Mg electrodes.


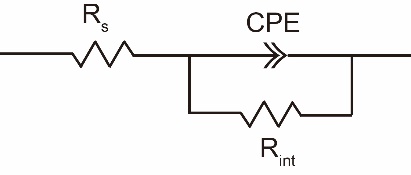


**Figure S5. Equivalent circuit model for fitting Nyquist plots of impedance measurements.** R(RQ), in which R is the resistance, CPE is the constant phase element.


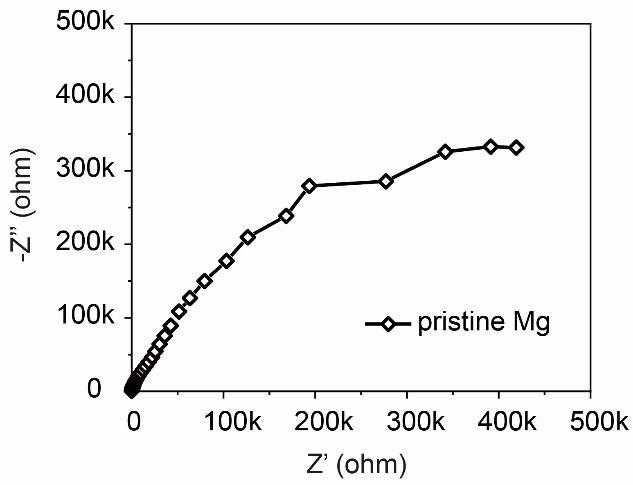


**Figure S6. Impedance spectroscopy of symmetric Mg cell with pristine Mg at room temperature.**


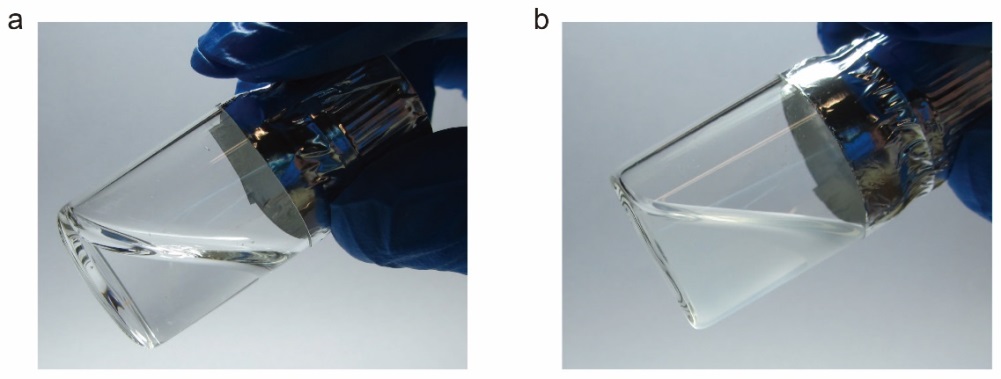


**Figure S7. Photos showing of 150 mM (a) and 160 mM (b) SnCl_2_-DME solution.**


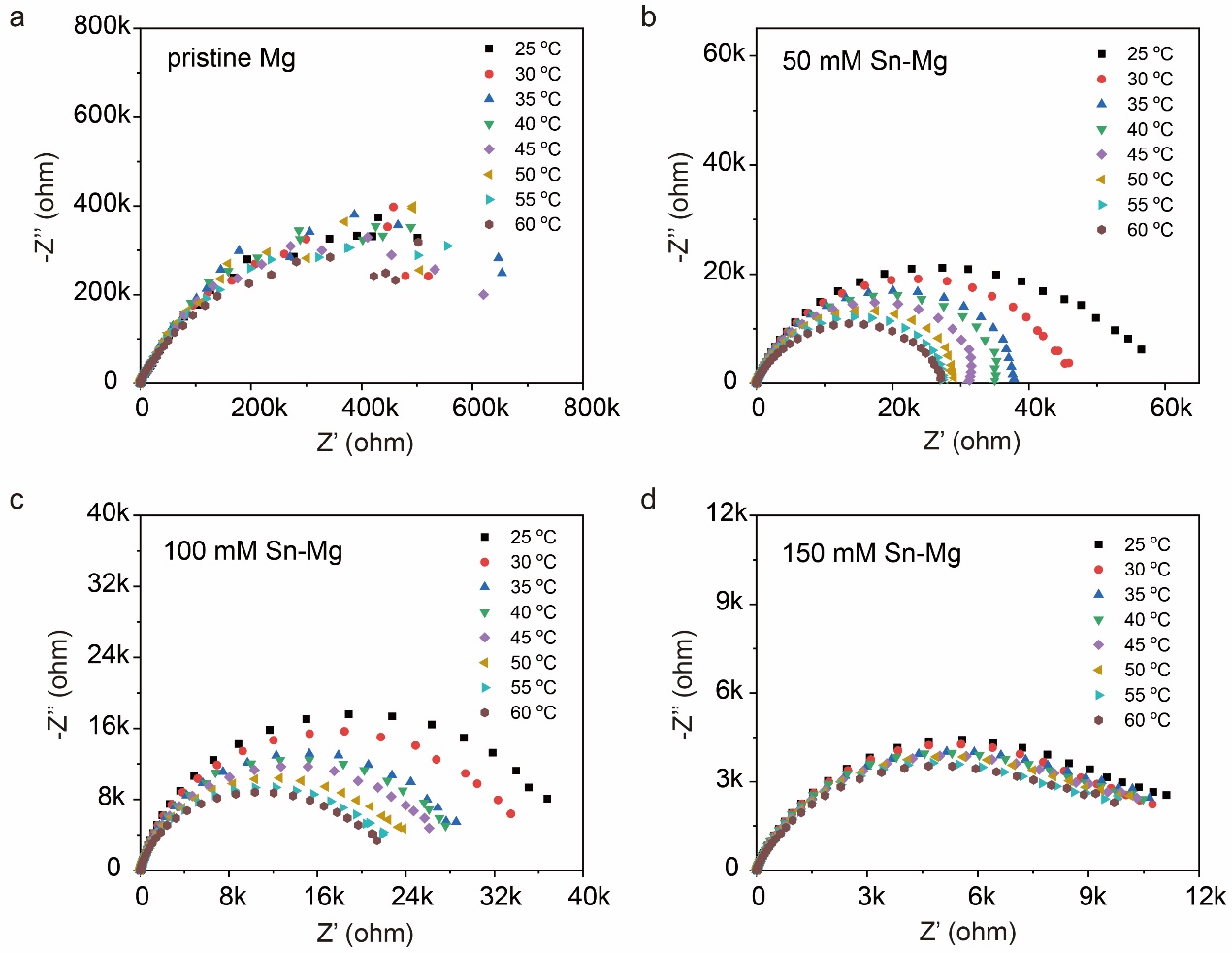


**Figure S8. EIS analysis temperature-dependent Nyquist plots for a symmetric Mg cell with pristine (a) and modified Mg electrodes prepared from SnCl_2_ solution with different concentrations of 50 mM (b), 100 mM (c) and 150 mM (d).**


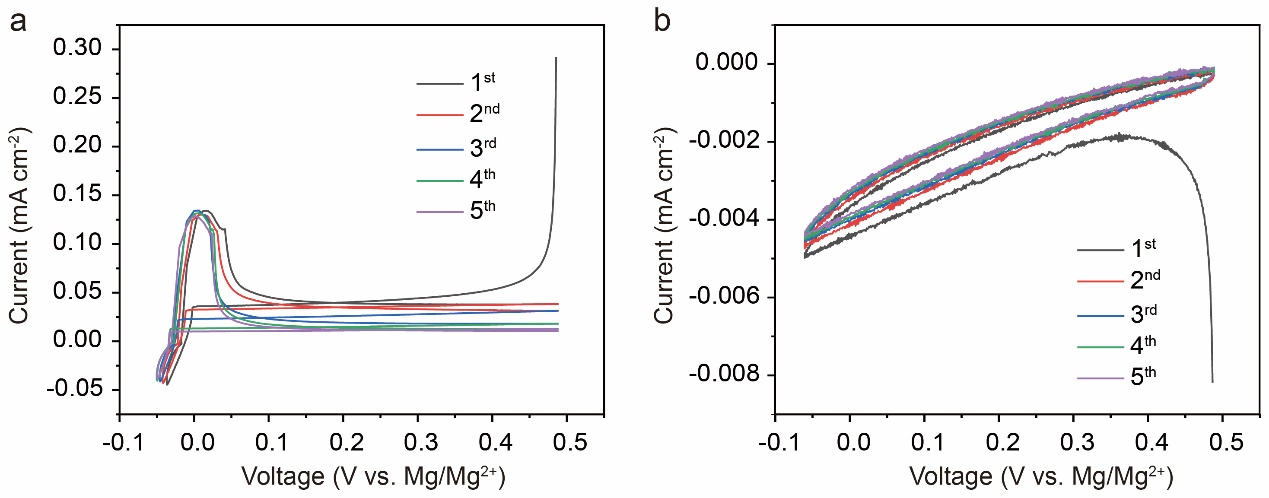


**Figure S9. CV curves of Mg plating/stripping using three-electrolyte system with a modified (a) and pristine (b) Mg working electrode, a modified and pristine Mg counter electrode and a Ag/AgCl reference electrode at 20 mV s^-1^.**


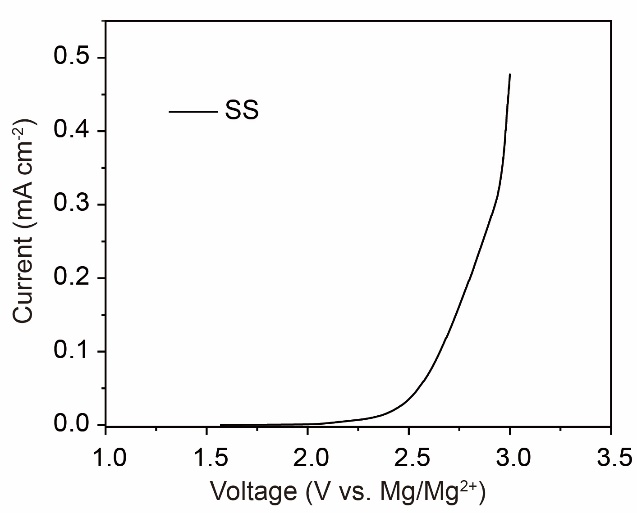


**Figure S10. LSV curves of the 0.5 M Mg(TFSI)_2_-DME electrolyte, scanning at a rate of 1 mV s^-1^ on a stainless steel (SS) working electrodes.**


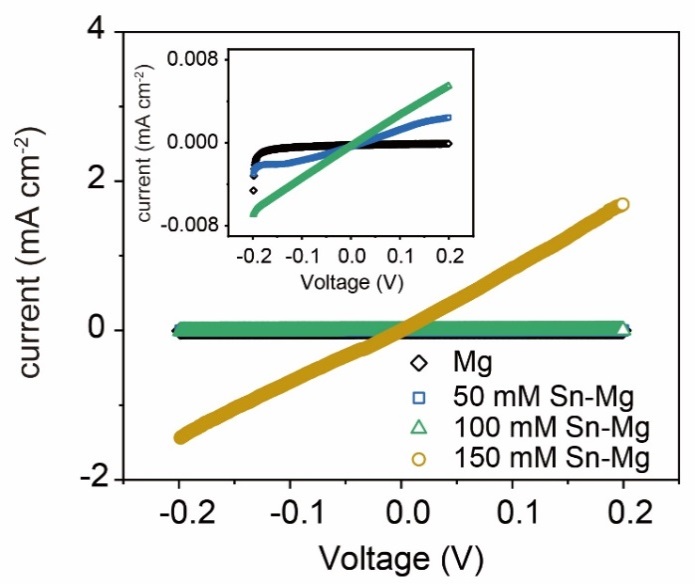


**Figure S11.** **CV curves of symmetric Mg cells with pristine and modified Mg electrode, at a sweep rate of 1 mV s^-1^ with the voltage range from -200 mV to 200 mV.** The insert is zoomed in segments of the symmetric Mg batteries with SnCl_2_-treated Mg anodes prepared from different concentrations of 50 mM (blue), 100 mM (green) and pristine Mg (black), separately.


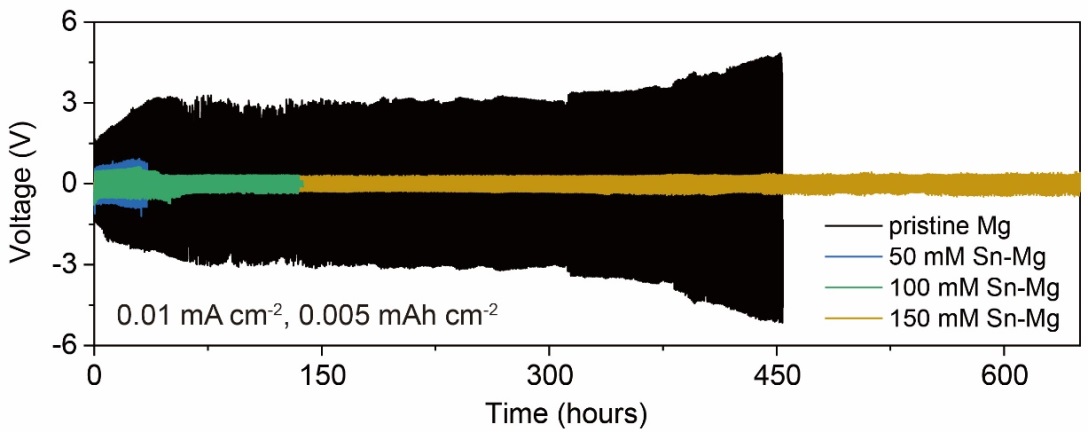


**Figure S12. Voltage profile of galvanostatic Mg plating/stripping on the symmetric Mg cells with pristine and modified Mg at a current density of 0.01 mA cm^-2^ for 0.5 h at each step.**


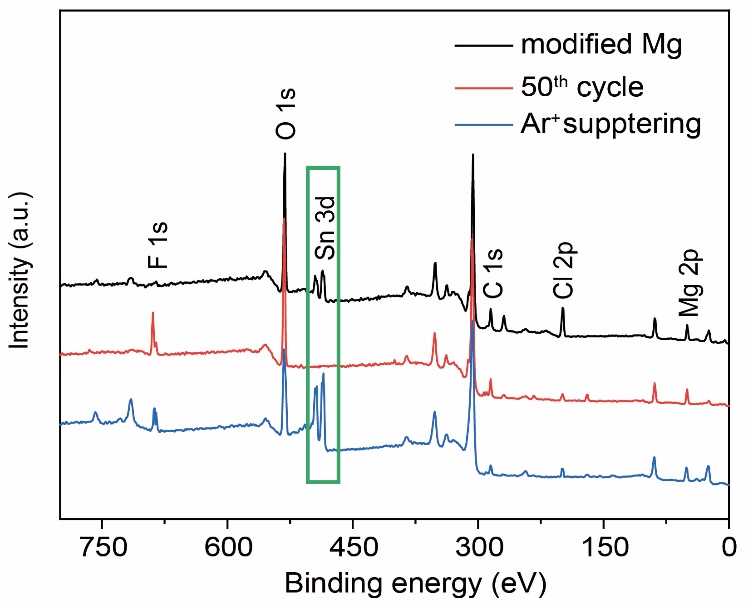


**Figure S13. XPS survey of the modified Mg metal anodes before cycling (black), and upon cycling for 50 cycles, before sputtering (red) and after 16 min Ar^+^ sputtering (blue), respectively.**


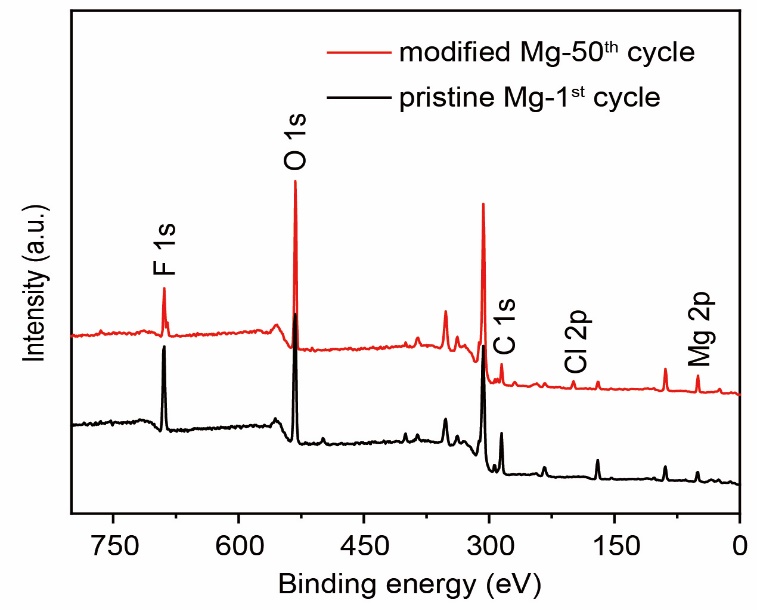


**Figure S14. XPS survey of the different samples upon cycling.** The modified Mg metal anodes for 50 cycles (red) and pristine Mg anodes for 1 cycle (black), respectively.


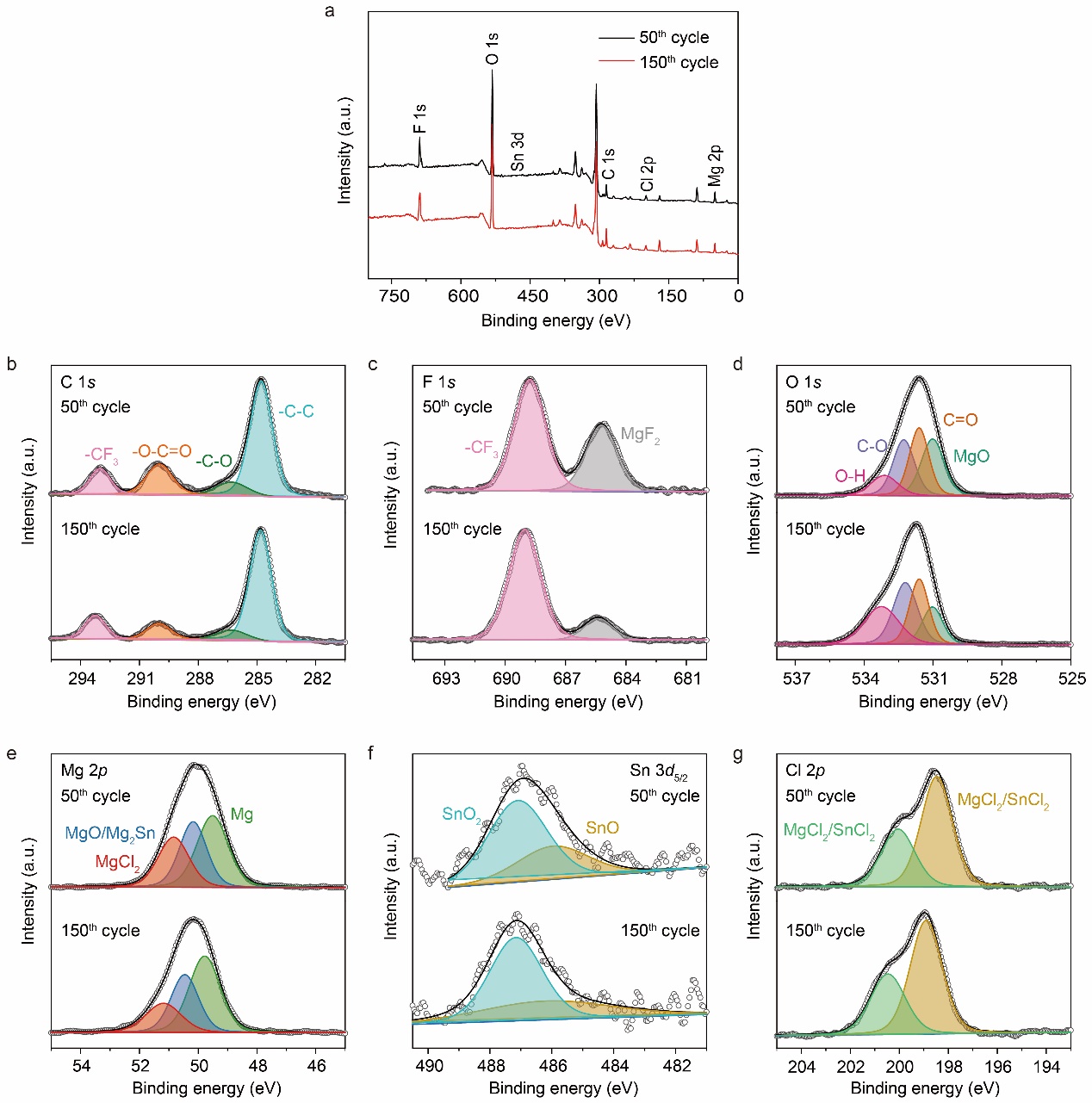


**Figure S15. XPS analysis of the modified Mg anodes upon cycling for 50 (top) and 150 (bottom) cycles.**


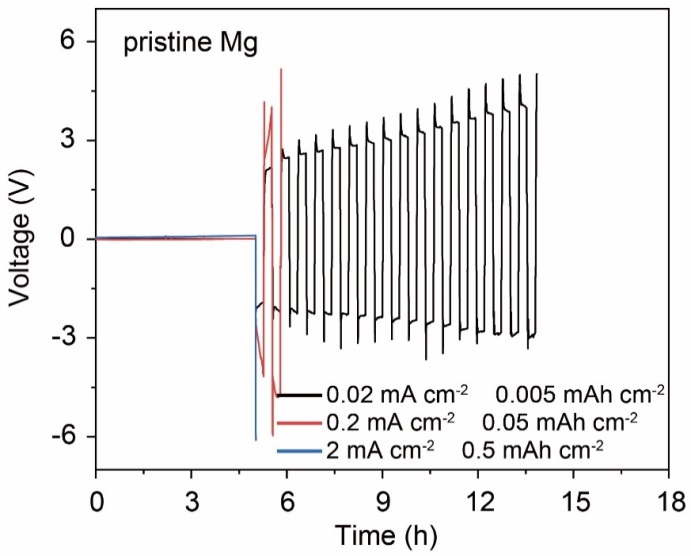


**Figure S16. Voltage profile of galvanostatic Mg plating/stripping on the symmetric Mg cells for pristine Mg electrodes at different current density.**


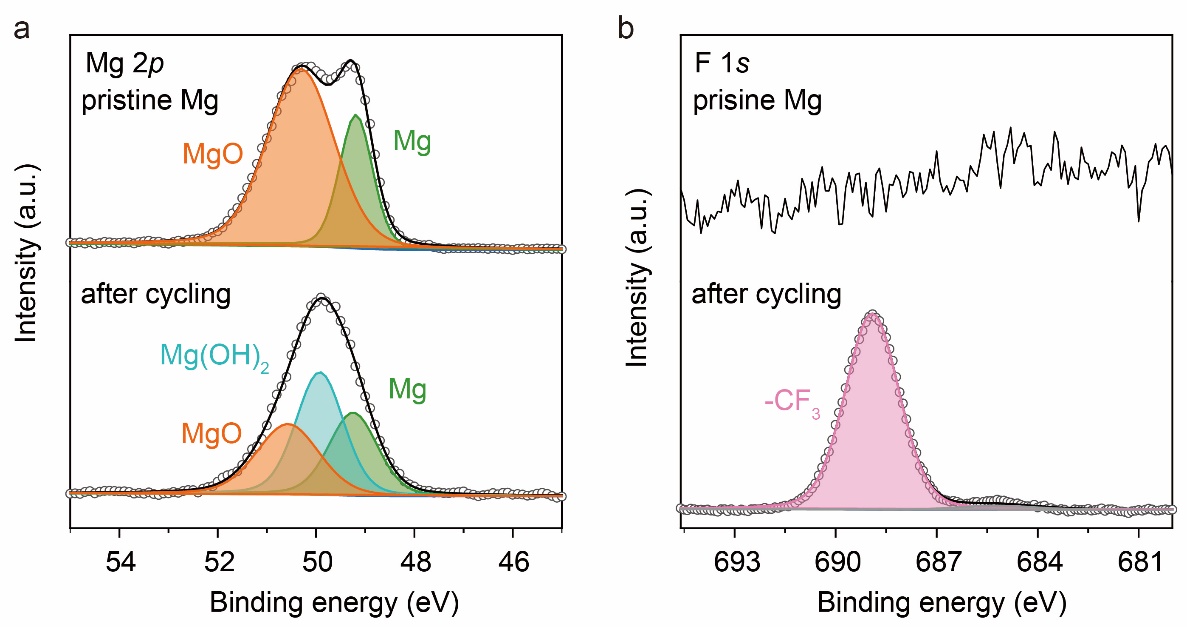


**Figure S17. XPS analysis of the pristine Mg anodes before and after cycling.** a) Mg 2p, b) F 1s of pristine Mg anode before (top) and after (bottom) cycling, respectively.


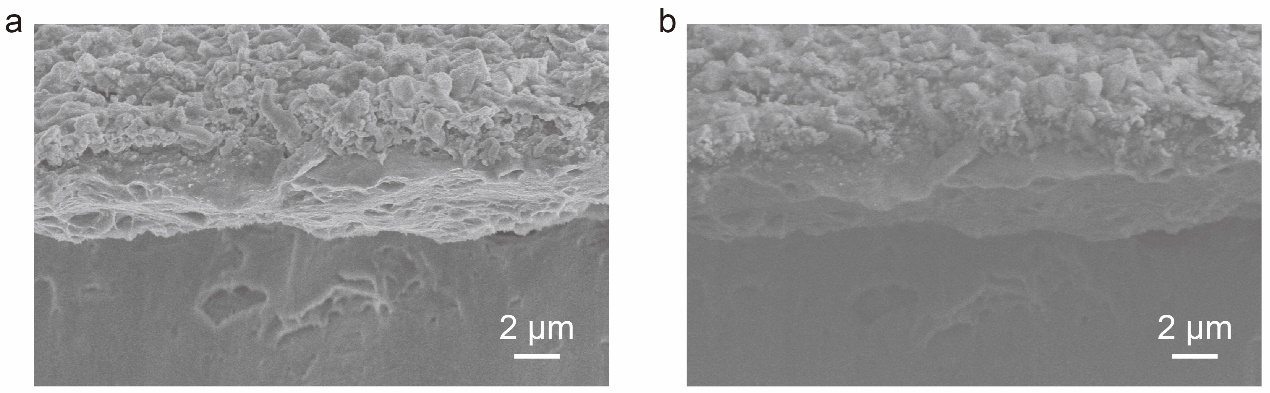


**Figure S18. Cross-section images of the modified Mg anode plated with 2 mAh cm^-2^ of Mg (a) and the corresponding image in backscattered electron mode (b).**


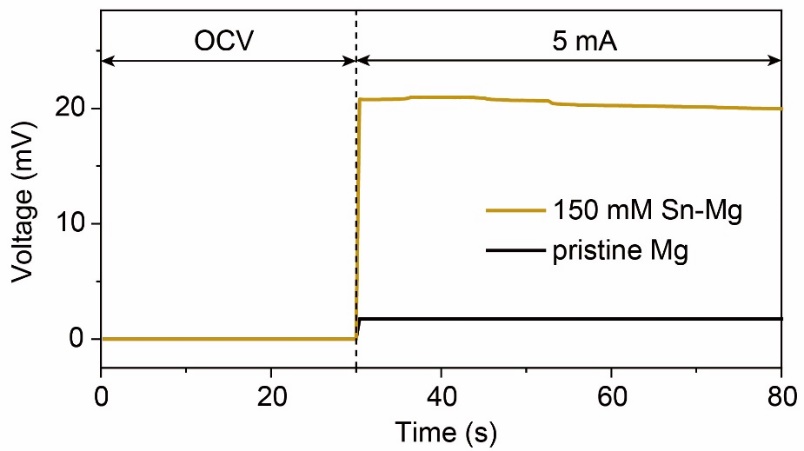


**Figure S19. Measurements of direct current conductivity of the modified (yellow line) and pristine Mg electrodes (black line).**


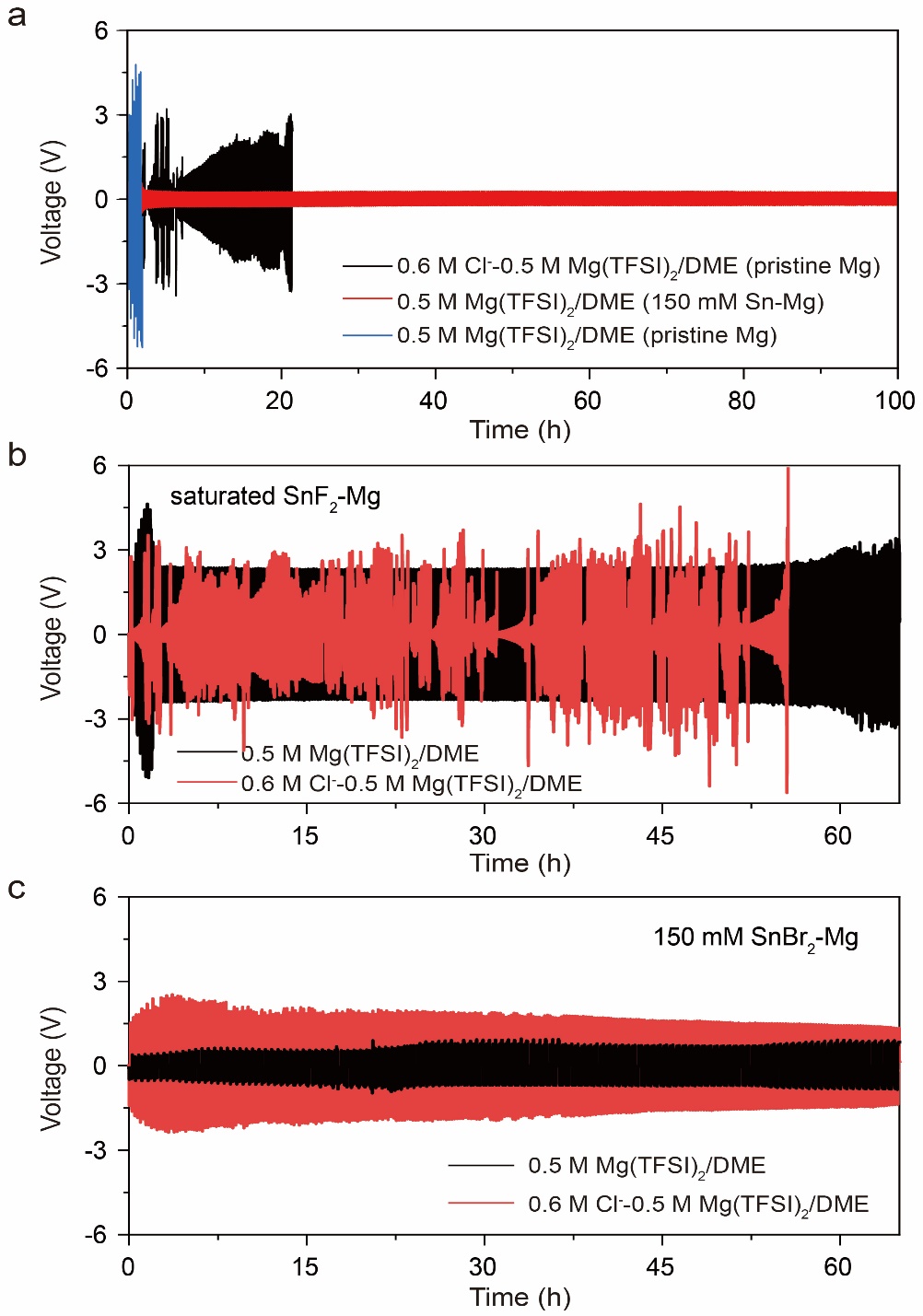


Figure S20. Voltage profile of symmetric cells with a) pristine and 150 mM Sn-Mg electrode, b) saturated SnF_2_-Mg electrode and c) 150 mM SnBr_2_-Mg electrode in different electrolyte at a current density of 0.05 mA cm^-2^.

**Table S1. The parameters obtained from the EIS curve fittings in the Figure S4.**

|  | Temperature (^o^C) | 25 | 30 | 35 | 40 | 45 | 50 | 55 | 60 |
| --- | --- | --- | --- | --- | --- | --- | --- | --- | --- |
| 50 mM Sn-Mg | R_s_ (Ω) | 2.504 | 2.383 | 2.326 | 2.275 | 2.23 | 2.199 | 2.169 | 2.146 |
|  | R_int_ (Ω) | 51822 | 44102 | 37753 | 35386 | 31905 | 28960 | 26927 | 25584 |
|  | Q (F) × 10^-6^ | 26.111 | 24.9 | 24.154 | 23.242 | 22.642 | 22.114 | 21.823 | 21.878 |
|  | a | 0.8825 | 0.88727 | 0.88965 | 0.89127 | 0.89288 | 0.89334 | 0.89197 | 0.88821 |
| 100 mM Sn-Mg | R_s_ (Ω) | 2.797 | 2.681 | 2.579 | 2.476 | 2.37 | 2.315 | 2.241 | 2.192 |
|  | R_int_ (Ω) | 41931 | 37193 | 31289 | 30166 | 28181 | 25169 | 23017 | 21964 |
|  | Q (F) × 10^-6^ | 85.175 | 85.436 | 85.548 | 83.647 | 82.052 | 81.831 | 81.692 | 80.096 |
|  | a | 0.87024 | 0.86886 | 0.86694 | 0.86615 | 0.86558 | 0.86261 | 0.85973 | 0.85833 |
| 150 mM Sn-Mg | R_s_ (Ω) | 2.372 | 2.239 | 2.05 | 2.125 | 1.975 | 1.926 | 1.868 | 1.831 |
|  | R_int_ (Ω) | 11364 | 10947 | 10676 | 10549 | 10287 | 10141 | 9640 | 9457 |
|  | Q (F) × 10^-6^ | 140.81 | 141.07 | 147.49 | 144.11 | 151.2 | 152.7 | 153.44 | 153.51 |
|  | a | 0.83206 | 0.83037 | 0.82835 | 0.83082 | 0.82585 | 0.8245 | 0.82352 | 0.82143 |
